# “We are not there yet”: perceptions, beliefs and experiences of healthcare professionals caring for women with pregnancy-related pelvic girdle pain in Australia

**DOI:** 10.1186/s12884-023-06000-x

**Published:** 2023-09-21

**Authors:** Dragana Ceprnja, Lucy Chipchase, Pranee Liamputtong, Amitabh Gupta

**Affiliations:** 1 School of Health Sciences, Western Sydney University, Sydney, Australia; 2 Physiotherapy Department, Westmead Hospital, Sydney, Australia; 3 College of Nursing and Health Sciences, Flinders University, Adelaide, Australia; 4 College of Health Sciences, VinUniversity, Hanoi, Vietnam

**Keywords:** Pregnancy, Pelvic girdle pain, Qualitative, Healthcare, Interviews, Medical, Midwifery, Physiotherapy

## Abstract

**Background:**

Pregnancy-related pelvic girdle pain (PPGP) is a common condition worldwide. Women report being unprepared about PPGP, and state they receive little recognition and support from healthcare professionals. Situated within the Common-Sense Model and Convergent Care Theory, this study sought to gain a conceptual understanding of the perceptions, beliefs and experiences of healthcare professionals who provide routine care for women with PPGP in Australia.

**Methods:**

A qualitative research design, using individual, semi-structured interviews with purposive sampling of healthcare professionals (*N*=27) consisting of doctors (*N*=9), midwives (*N*=9) and physiotherapists (*N*=9). Most participants were female (22/27) with a range of professional experience. An interview guide consisting of open-ended questions was used with a flexible and responsive approach. Thematic analysis was performed where interview data were transcribed, coded, grouped into meaningful categories and then constructed into broad themes.

**Results:**

Four themes were identified: 1. Identity and impact of PPGP; 2. What works well?; 3. What gets in the way?; and 4. Quality care: What is needed? Healthcare professionals recognised PPGP as a common and disabling condition, which created a large impact on a woman’s life during pregnancy. Stepped-level care, including education and physiotherapy intervention, was seen to be helpful and led to a positive prognosis. Barriers at patient, clinician and organisation levels were identified and led to consequences for women with PPGP not receiving the care they need.

**Conclusion:**

This study elucidates important implications for health care delivery. Acknowledging that PPGP is a common condition causing difficulty for many women, healthcare professionals identified strong teamwork and greater clinical experience as important factors in being able to deliver appropriate healthcare. Whilst healthcare professionals reported being committed to caring for women during pregnancy, busy workloads, attitudes towards curability, and a lack of formal education were identified as barriers to care. The findings suggest timely access, clear referral pathways and an integrated approach are required for best care practice for women with PPGP. A greater emphasis on the need for multidisciplinary models of care during pregnancy is evident.

**Supplementary Information:**

The online version contains supplementary material available at 10.1186/s12884-023-06000-x.

## Background

Pregnancy-related pelvic girdle pain (PPGP) is a common musculoskeletal condition with a prevalence of 44% in Australia and ranging from 7% to 84% globally [1–5]. A large proportion of women with PPGP report moderate to severe pain along with a diminished ability to perform everyday activities, such as getting up from a chair, bending and walking [6, 7]. Although the pain can commence anytime during pregnancy, a significant proportion of women continue to report symptoms following childbirth and beyond [2]. Consequently, PPGP has been considered a major public health issue and warrants effective management [8]. However, women describe feeling unprepared for the impact of PPGP and not receiving enough recognition or support from healthcare professionals (HCPs) [7–13]. These findings demonstrate there is a mismatch between the large numbers of women affected and the level of care that is afforded to manage PPGP.

This gap in care may be related to the management of PPGP not being widely understood by HCPs. To some degree, this is supported by a recent study which reported that Irish physiotherapists believed PPGP to be a complex clinical presentation requiring early detection and shared non-evidence based perspectives related to biomechanics and pelvic stability [14]. Similarly, a qualitative study in Sweden reported that midwives considered PPGP to be a common clinical problem requiring support, however their knowledge was self-acquired through personal experiences, suggesting a need for more training and education [15]. Much like other conditions, knowledge translation efforts, including practitioner training to support the provision of evidence-informed care that aligns with clinical practice guidelines, are needed [14, 16].

While knowledge is vital, other factors have the potential to impact on the provision of care for women with PPGP. This includes the beliefs and attitudes of HCPs towards PPGP. There is evidence that some HCPs regard pelvic girdle pain as normal during pregnancy and believe it does not warrant intervention [7, 15]. Such beliefs may reduce the amount of attention HCPs provide to women or result in a lack of empathy. A dismissive attitude in maternity care has been shown to reduce patient satisfaction and undermine patient-provider interactions [17]. This may explain why women with PPGP often report a lack of support from HCPs and seek more recognition [9, 10].

Adequate time for healthcare consultations is also recognised as an integral component of maternity care to meet care needs [17, 18]. However, a lack of time in busy clinical settings may be a barrier to providing care to women with PPGP, as suggested in a single Swedish study [15]. It is plausible that this may be a problem in Australian settings too, however, more information is needed to elucidate what HCPs view as the main impacts to care provision for PPGP. Therefore, this study aimed to determine HCPs’ beliefs, perceptions, and experiences of PPGP, and their views about how it should be managed during pregnancy.

### Theoretical frameworks

This study utilised a theory-driven approach for interpreting and representing findings and was situated within the Common-Sense Model (CSM) and Convergent Care Theory (CCT) [19–21]. These theoretical lenses allowed for the examination and interpretation of the responses from HCPs which arose from complex and real-world clinical settings.

The CSM of self-regulation of health and illness is a well-established theory that has been used extensively in health research to explore relationships between cognitive illness representations and health behaviours [20]. Central to the CSM are representations, or beliefs, that people have about illness. Leventhal and colleagues (2003) described five elements of these representations: identity (the label or name given to a condition); cause (ideas about perceived causes of a condition); consequences (perceptions regarding the consequences of a condition); timeline (beliefs about how long the condition will last); and curability (beliefs about the extent to which a condition can be cured or controlled) [20]. Previous studies have adapted the CSM to explore beliefs about a condition or issue from those affected indirectly such as family members and HCPs [22, 23]. Therefore, in the context of the current study, the CSM was adopted to investigate HCP’s beliefs about PPGP and was used to design the research questions, organise the interview schedule and guide the analysis process (Table 1).

**Table 1:** Interpretation of CSM theory

Domain	Original understanding	Interpretations in this study
Identity	The label given to a condition	The label given to PPGP and identification of how PPGP is seen and perceived
Cause	Ideas about perceived causes	Ideas about perceived causes and/or risk factors for PPGP
Time-line	Beliefs about how long the condition will last	Beliefs about how long PPGP will last and temporal aspects about the management of PPGP, such as the best time to intervene
Curability	Beliefs about the extent to which a problem can be cured	Beliefs about the extent to which PPGP can be cured
Consequences	Perceptions regarding the consequences and impact of a condition	Perceptions regarding the consequences and impact of PPGP

The CCT underpins the caring culture that strives to unite healthcare stakeholders, bond resources, and join forces to achieve optimal healthcare outcomes [21]. Based on the empirical evidence and practice models, CCT encompasses four key concepts including: organisational care (positive environment, culture and support system that empowers team members); collaborative care (HCPs with various professional backgrounds work together to provide best optimal care); precision care (person-centred care tailored to meet individual patient’s care needs); and self-care (individuals take care of themselves by actively engaging in healthy behaviours and activities) [21]. While the CCT is relatively new and has limited supporting evidence, the origins of it are grounded in clinical practice. The CCT establishes the need for all stakeholders, particularly clinicians, to work collaboratively to achieve best care incorporating the growing awareness of the important role of patients’ involvement in their own self-care [21]. Considering the perspectives of HCPs in this study, the CCT provided an innovative framework towards improving the management of PPGP in ante-natal care.

## Methods

### Design and setting

This study adopted a qualitative descriptive design using semi-structured interviews to obtain an in-depth description of the beliefs and perceptions of HCPs on PPGP [24]. A qualitative approach was essential because little is known about the experiences of HCPS managing women with PPGP.

This study was conducted at a hospital in Sydney, Australia from August 2021 to August 2022. The hospital is a large teaching and tertiary referral government funded hospital in an urban centre with over 5,200 births recorded annually [25]. The hospital has inpatient and outpatient services for maternity care, including ante-natal and post-natal services. Ethical approval was granted by the institutional Human Research Ethics Committee. The checklist for the consolidated criteria for reporting qualitative research (COREQ) was adhered [26].

### Participants

Purposive sampling method was used to recruit doctors, midwives and physiotherapists who provided routine ante-natal care and could provide information rich data, relevant to the study aims [24]. It is of note that not all HCPs work in the same clinical setting. Whilst the doctors and midwives work in the ante-natal clinic, the physiotherapists work in an outpatient setting or with inpatients on the maternity ward where they may manage women with PPGP as part of their caseload. Information about the study was distributed via email and posters in the ante-natal clinic, and HCPs interested in participating were directed to contact the researcher (DC) by email or phone to register their interest. The lead researcher (DC) provided potential participants with verbal and written information about the study and discussed any questions raised. Health care professionals were assured that confidentiality and privacy would be maintained and were also informed that they could withdraw from the study at any time without their employment or relationship with the hospital being affected.

### Sample size

The sample size required was determined when saturation of themes was achieved such that the collection of new data did not add any further information on the aims of the study [24]. It has been suggested that a minimum sample of six participants is required when analysing interviews with the focus on identifying patterned meaning across cases rather than idiographic meaning within cases [27]. Thus, it was planned that at least nine participants from each professional group of doctors, midwives and physiotherapists would be interviewed to ensure richness of the data with a broad and diverse sample. The enrolment of HCPs within each of the groups was split and matched for early-career, mid-career and experienced professionals. Although saturation of themes was evident after the fifteenth interview, the decision was made to continue to collect data as planned from 27 participants to further probe and explore themes to be certain that no further information became available with more interviews and to allow for deeper understanding [24].

### Procedure for interviews

The first author (DC) contacted each participant to schedule an interview either in-person or via tele-video. Participants were asked to report their age, profession, and years in practice. An interview guide consisting of open-ended questions ensured that rich data about the topic was obtained (see Additional file 1). The interview guide incorporated elements of the CSM and additional questions to allow for flexibility to uncover other concepts. All participants were asked about their perceptions of PPGP, experiences with management, their confidence caring for women with PPGP, and expectations about health care delivery for this condition. The interview guide ensured the same range of topics was discussed within each individual interview.

The interviews were digitally recorded, and all participants were assigned a coded number to protect their identities. Ranging from 45 to 60 minutes duration, the interviews were conducted by the same researcher (DC) and transcribed verbatim directly after completion. The transcript was then provided to participants, referred to as a member checking method [24]. The method ensured that participants were able to review and edit their responses if they felt more information was needed or to reword text if they were not comfortable with it being included and to ensure the accuracy of their views and experiences.

### Data analysis

Thematic analysis with a mixed deductive and inductive approach was used to analyse interview responses [24]. The CSM was used to guide analysis deductively and the authors remained open to other themes developed through an inductive approach. Firstly, each recorded interview was listened to several times to make sense of the data and the interview as a whole [24, 28]. Open coding was conducted by naming sections of the participants’ narratives in the text [29]. Subsequently, there was a regular discussion between all authors to ensure thorough and consistent coding patterns of the interview data. In the process of coding, it became clear that the CSM did not account for all the emerging codes, therefore some inductively derived codes were added to label this information. To enhance trustworthiness, codes were then grouped to form meaningful categories as agreed upon by all authors. The next step was to construct the final framework of broad themes from the categories with discussion and comparison amongst all authors, moving back and forth between text and categories to enrich the credibility of the data.

### Reflexivity

There was a strong commitment from the authors to work collaboratively in the collection, analysis, interpretation and reporting of the qualitative data with different levels of involvement at each stage of the study. All the interviews were conducted by one researcher (DC) who is a registered and experienced physiotherapist. The primary researcher’s interest in the topic, having previously worked in ante-natal care, may have enabled the participants to talk openly about their experiences. In order to avoid personal biases, the progress of fieldwork and interviews were regularly discussed among the research team. Input was sought from all members of the research team, in particular PL has extensive experience in the collection and analysis of qualitative data. Data analysis drew on the combined insights of all members of the research team.

## Results

Twenty-seven health care professionals participated in this study, with nine doctors, nine midwives and nine physiotherapists of a broad range of age and professional experience in ante-natal care (Table 2). Most participants were female (22/27). Eleven interviews were conducted in-person and 16 interviews were conducted via tele-video, based on participant preference.

**Table 2:** Participant characteristics (*N*=27)

Participant	Profession	Gender	Age (years)	Ante-natal experience
1	Doctor	Female	26-35	6-10 years
2	Physiotherapist	Female	<25	<6 months
3	Physiotherapist	Female	26-35	6-10 years
4	Doctor	Female	26-35	6-10 years
5	Doctor	Female	26-35	1-2 years
6	Doctor	Female	26-35	3-5 years
7	Physiotherapist	Female	<25	<6 months
8	Physiotherapist	Female	26-35	3-5 years
9	Physiotherapist	Male	26-35	6-10 years
10	Midwife	Female	56-65	>11 years
11	Midwife	Female	56-65	>11 years
12	Midwife	Female	26-35	6-10 years
13	Physiotherapist	Female	26-35	3-5 years
14	Doctor	Male	36-45	6-10 years
15	Midwife	Female	56-65	>11 years
16	Doctor	Male	56-65	>11 years
17	Doctor	Female	36-45	>11 years
18	Physiotherapist	Male	<25	<6 months
19	Physiotherapist	Female	<25	1-2 years
20	Physiotherapist	Female	36-45	>11 years
21	Doctor	Female	<25	1-2 years
22	Doctor	Male	<25	3-5 years
23	Midwife	Female	26-35	6-10 years
24	Midwife	Female	26-35	3-5 years
25	Midwife	Female	46-55	>11 years
26	Midwife	Female	36-45	>11 years
27	Midwife	Female	56-65	>11 years

Four themes were identified: 1. Identity and impact of PPGP; 2. What works well?; 3. What gets in the way?; and 4. Quality care: What is needed? Verbatim quotes from the interviews are presented to support these themes using the assigned participant code number and profession.

### Identity and impact of PPGP

Health care professionals concurred that PPGP was common during pregnancy and estimated up to half of all pregnant women were afflicted with some degree of pain. Despite this view, most HCPs in ante-natal care remarked that they did not routinely ask women about the presence of pain and acknowledged that PPGP was almost certainly under-reported.

*“I think you would find higher numbers if we were asking all women about pain, but it is not usual practice. Mainly I will ask women [only] if they look like they have pain” (Midwife 9).*

The pain was described in patho-anatomical terms by HCPs as being in the pelvic region and attributed to forces and loads related to changes in the body due to pregnancy. However, many HCPs across each of the professions acknowledged the influence of psychosocial factors on PPGP, particularly in the context to how women coped with the pain.

*“Some women get this pain and then seem to cope with just some discomfort, like annoyance. Other women get really distressed with it, out of proportion, like really over-react” (Midwife 1).*

Significant physical impacts were perceived by HCPs as affecting all aspects of a woman’s daily life when suffering from PPGP. They identified mobility issues, reduced capacity for exercise and household chores, and challenges with paid employment. Considering both the physical and psychosocial impacts, HCPs believed that women with PPGP “*struggle and have a much harder pregnancy*” (Midwife 23).

Many HCPs spoke of seeing women who were completely unprepared for the severity of symptoms. These women were *“completely miserable”* and often accompanied to appointments by partners and/or family members who were also *“very distressed”*. Where pain management as an inpatient was required, HCPs regarded this as a very stressful situation for all involved. Women with pain worried about how they would cope with childbirth and looked to HCPs for support. A few HCPs described situations where women were so unhappy and distraught that they requested induced labour to be rid of the pain. In a few circumstances, labour was induced early because of PPGP.

*“I’ve seen one case where a woman was not quite [full] term, who genuinely got to the point where she couldn’t walk … we ended up inducing her quite early just so she could not be pregnant anymore” (Doctor 1).*

There was the belief that PPGP did not just affect the pregnancy experience but continued beyond to have broader negative impacts on women’s lives. For example, one midwife stated: *“This bad pregnancy experience colours so much about life afterwards and you have to look at the whole picture”* (Midwife 10).

### What works well?

Most HCPs suggested that women who received treatment for PPGP usually experienced a tangible benefit in their symptoms. Health care professionals remarked that women were appreciative of information about the condition, including tips regarding self-management. For some women, mainly those with mild symptoms, education and advice was considered by HCPs as sufficient to meet care needs. Women with severe symptoms, generally with greater levels of pain and disability, were perceived to require a higher level of care and were often referred to physiotherapy.

*“Most women benefit from some simple education, advice and reassurance. If this is not sufficient, or they are clearly very bothered by the pain and can’t cope, then I will refer them to physiotherapy” (Doctor 17).*

Physiotherapists adopted a multi-modal approach to management and were of the view that they “*delivered good care*” to women who “*responded well*” to physiotherapy interventions (Physiotherapist 13). This was supported by the views of doctors and midwives who acknowledged the beneficial effect of physiotherapy.

*“You know the patients are very satisfied with physiotherapy care, when they are referred and see physiotherapy they are very happy with their care” (Midwife 14).*

Some women were also referred by midwives and physiotherapists to doctors for help with medications or if a medical review was deemed necessary. Thus, an individualised approach was adopted where women were provided with options for management according to their perceived needs. A doctor remarked: *“Sometimes it may just be education or information, sometimes it is exercise, sometimes seeing the doctor, maybe medication, maybe physio, maybe hydrotherapy … essentially options that can help”* (Doctor 17).

Most HCPs discussed the positive effects of intervention; however, a few believed PPGP was not “*curable*” and would persist for the duration of pregnancy. These HCPs spoke of women needing to endure the pain until the baby was born: *“Unfortunately it’s something that we can’t really cure, we kind of need to let it play out for the duration of the pregnancy”* (Physiotherapist 8). However, the majority of HCPs regarded PPGP as *“manageable*” if early access to the appropriate care was provided. They spoke of the importance of early identification of women with PPGP to facilitate timely interventions, allowing women to “*get on top of the pain*” by following recommended advice and exercise. The provision of support and reassurance, delivered with empathy, was considered essential to build self-efficacy and empower women to cope with PPGP.

Health care professionals agreed that on-the-job training and strong support from senior colleagues helped them provide good care to women with PPGP. The experience of HCPs was perceived as an important factor in being able to deliver care. Within each profession, senior HCPs felt *“confident”* managing women with PPGP. In contrast, junior staff felt *“less prepared”* and *“less comfortable”*. However junior staff discussed the strong culture of teamwork in maternity care settings, where senior colleagues were keen to share tips and advice about PPGP management. Some HCPs identified the need to provide basic training about PPGP for all new staff.

The availability of good resources, in written and electronic forms, was identified as a beneficial factor in being able to deliver appropriate care to women with PPGP. This was described as being able to ensure consistency of information across professions and reducing mixed or conflicting messages about PPGP. However, not all HCPs were aware of the existence of the resources or where to locate them. Some HCPs reported that better visibility of resources was warranted and could be achieved by adding information to orientation and induction programs: *“Making sure all staff know what resources are available as we throw a lot of education their way”* (Physiotherapist 18).

### What gets in the way?

To allow for a deeper exploration of factors impeding care provision for women with PPGP, three subthemes were created, including: a system in crisis; clinicians downplay pain; and the struggle with culturally safe practices.

#### A system in crisis

The fast-paced antenatal setting in a tertiary, teaching hospital with heavy caseloads was seen as the major barrier to HCPs providing the necessary care for women with PPGP. Doctors and midwives spoke of time constraints, which limited the ability to identify women with PPGP and affected care provision.

*“(Care) is limited by how busy we are which means we don’t have enough time to do what we want … Sometimes you are scared to ask women if they have any questions because you just don’t have the time to spare to answer anything that is not urgent. Sounds terrible I know, but it is the honest truth” (Midwife 26).*

Midwives and doctors reported they did not prioritise pain when there were competing demands in their clinical duties. This resulted in less *“airplay”* about PPGP and was attributed as being due to time constraints rather than clinicians’ attitudes towards PPGP.

*“It is not that staff want to ignore women, it is just that time is short and you are focussed on getting through what you need to do for that appointment” (Doctor 17).*

Many doctors and midwives commented on problems related to being able to refer women to physiotherapy. They were confused about whether there was a paper or electronic system, and some considered this extra step (or steps) a challenge with competing demands in ante-natal care. Many were held the opinion that there were long waiting times for women to access physiotherapy and lamented that not having a physiotherapist in the ante-natal unit caused unnecessary delays in women accessing early assessment and intervention for PPGP: *“If you had physio in clinic it would eliminate the wait”* (Midwife 10).

Physiotherapists, on the other hand, spoke of receiving referrals that lacked sufficient details, causing interruptions or delays to contact women with PPGP to schedule appointments. They reported variability in information included as there was not a standard referral template available. Physiotherapists were also frustrated with referrals being made late in the third trimester, often at 38 weeks’ gestation or beyond, seeing this as a missed opportunity for early intervention.

*“It is quite upsetting when we get these referrals and they are quite late in the pregnancy and they’ve been having pain for months. When they could have been given this education and exercises much earlier” (Physiotherapist 3)*.

The nature of ante-natal clinics where women are seen by a different clinician each visit was also identified as a health service factor affecting care provision. The lack of continuity of care and professional rapport, often built over time, meant there was the chance that women may *“fall through the cracks”.* The notion that care may not be co-ordinated in the best way possible at a health service level was a consistent narrative amongst all groups of HCPs. One physiotherapist elaborated:

*“At the moment it can be hit and miss depending on who you see and what referrals are made. So inevitably and unfortunately, there are women missing out on having the care they need in the current system” (Physiotherapist 19).*

#### Clinicians downplay pain

Despite the overall impression that the management of PPGP formed part of routine care, some HCPs raised the possibility that there are clinicians who are dismissive of the pain and treat PPGP as a normal part of pregnancy. A midwife remarked that *“I think there are a few clinicians that are very dismissive, you know this is pregnancy, you will have pain, there is an element that you just have to put up with it”* (Midwife 10). Doctors in particular thought their medical colleagues may not pay PPGP the attention it deserves: “*I honestly think most of my colleagues would just ignore it”* (Doctor 4). A similar view was expressed by some midwives and physiotherapists who suggested they thought women “*may be missed if they are only seeing doctors*” (Physiotherapist 7).

The level of knowledge of HCPs was identified as a factor that influences care provision by all three professions. It was postulated that HCPs with less experience may not be able to provide comprehensive care compared to those with more experience due to a lack of knowledge about the condition and understanding of treatment options available. Further, according to many, PPGP was not included in their undergraduate training, and they suggested more education of new and junior staff to build workforce capability. Few reported having attended external courses or professional development opportunities relating to PPGP.

#### The struggle with culturally safe practices

There was a common theme amongst HCPs that some women did not cope well with PPGP due to a range of barriers. A lack of motivation was identified, and HCPs reported that despite receiving education, many women did not follow up on the advice to exercise or follow through with recommendations to use a pelvic belt or gait aid to help manage pain. Whilst some HCPs felt this was because the women *“did not listen”*, others perceived it as a *“reluctance to make changes”* or, more simply, *“not wanting to actually do anything”* to help themselves.

*“They will complain about it but when you say there are things you can do then women may not actually do those things. So they won’t get better because they’re not doing what they need to do” (Doctor 1).*

Hence, HCPs reported a strong emphasis on managing the patient’s expectations and promoting self-management strategies. There was an agreement between HCPs that interventions should be matched to the woman’s goals. *“No point talking about exercise if the person isn’t interested … so then it is about matching what they are willing to do with what we can provide”* (Midwife 23).

Engaging women from culturally and linguistically diverse (CALD) backgrounds was viewed as a challenge by HCPs in this study. There was the notion that women from certain CALD backgrounds may be more prone to catastrophizing pain behaviours and less willing to exercise due to existing cultural beliefs.

*“Sometimes it may be related to their culture and they don’t do exercises in pregnancy. So it is impossible to get them to do any exercise when they have a whole family at home telling them to stay off their feet. You can’t win” (Midwife 26).*

However, there was an acknowledgement that women from CALD backgrounds may be less empowered to discuss PPGP in the health care setting and so may be *“quietly suffering”*. One doctor stated: *“Some women from certain cultural backgrounds may feel intimidated about complaining to doctors about how they feel … or they may not feel empowered to do this”* (Doctor 17). Ensuring resources are available in a range of languages and investing in multicultural health support were seen as important strategies to help improve access to care for women from CALD backgrounds.

Other patient factors mentioned by HCPs included women of younger age and those who could not prioritise time for their own health needs due to competing demands, such as employment, childcare and family commitments. These groups of women were seen to *“have a much harder time dealing with pain”* due to limited engagement and participation in health care despite the best intentions of HCPs.

*“When they’ve got many kids, competing priorities and demands and they can’t always put themselves first … I find that sometimes quite hard to work around … even though we try and problem solve this if there is no change that occurs around their routine then that’s a bit tricky I find”* (Physiotherapist 20).

### Quality care: what is needed?

There was a strong view amongst HCPs that the current healthcare system failed to meet the needs of all women with PPGP and that there is scope for improvement. Health care professionals had views on elements of care they felt were essential with similar suggestions from the three professions (see Table 3). A clear and consistent message was the need for multidisciplinary and integrated care to manage PPGP effectively in the ante-natal setting. Midwife 10 said: *“I would love to be able to say you are in the best place where all of your health care needs are looked after in clinic, but we are not there yet”*.

**Table 3:** Essential elements of integrated care for women with PPGP as identified by HCPs

Suggestion	Quote
Screening for PPGP	*“More proactive in asking the women about pain rather than just waiting for women to mention it themselves ... then we may be able to catch women earlier” (Physiotherapist 13)* *“Screening early to be able to target the risks before the pain takes hold” (Midwife 15)* *“Access to early screening. Here could be a focus on women with risk factors, for example multi-parity, or those in third trimester where there is higher risk of having PPGP” (Doctor 16)* *“Screening women so we don’t miss them and don’t miss the opportunity to intervene early” (Physiotherapist 20)*
Education resources	*“More information, more education and maybe cultural and language specific” (Doctor 1)* *“Be pointed in the right direction that there are resources on the webpage and education sessions they can attend” (Physiotherapist 13)* *“I think it comes down to education, education, education and early” (Midwife 15)* *“We need more resources translated into different languages” (Doctor 5)* *“There is the scope with telemedicine to have online information sessions that can reach a large number of people without the need to come into hospital” (Doctor 22)*
Individualised treatment options	*“Having some online videos may also be flexible, so people can use them as needed” (Midwife 12)* *“For all women to have access to the services they need, whether that is fact sheets and brochures online, or hydro classes or physiotherapy. Just more flexibility in what we offer and when to match their needs” (Midwife 12)* *“I think it is about all women having access to the care they need, whether that be access to written information, a quick education session by the doctor or midwife in clinic, or referral to physio as needed” (Physiotherapist 20)* *“Women seem to be able to learn how to cope with pain better when they understand what options they have in being able to try things to help the pain” (Physiotherapist 9)*
Multidisciplinary teams	*“There is a need to have more allied health in the clinic. There are many people who can help with a piece of the puzzle” (Midwife 10)* *“We need links to services to better support women, it is not up to the midwife alone to fix this” (Midwife 11).* *“At the moment, we send them to physio, which is somewhere down the back from the hospital, why can’t we have physio here in the clinic where it is needed?” (Doctor 14)* *“I think best care should be multidisciplinary so that it can meet individual needs” (Physiotherapist 19)* *“Ensuring there is a multi-disciplinary team that is dealing with things per their scope” (Doctor 22)* *“It is about having the right staff mix to deal with this” (Midwife 26)*

The idea of a *“one-stop shop”* was mentioned to reduce the fragmentation of care and facilitate multidisciplinary input including midwifery, medical and allied health services in one central location. Having physiotherapy in the ante-natal clinic was viewed as a solution to streamline access, avoid unnecessary delays, and reduce variations in care. Included in this model could be a routine screening of all women and a decision-making tool to support timely and appropriate referrals to physiotherapy.

“*I think the midwives could ask all women coming for their 28 week (appointment) about pain and then provide information or a referral to physio as needed” (Physiotherapist 2).*

Health care professionals spoke of experiences in other settings where integrated models were successful and that ante-natal services could learn from these exemplars. Mainly, the ability to individually tailor interventions from a suite of options according to patient needs was viewed as an important aspect of high quality care. *“I mean look at areas like cancer care where there are these fantastic multidisciplinary models and they can link in to services they need. That’s how we should be running our services”* (Midwife 10). Adopting a multidisciplinary approach was also seen by HCPs as a way of building shared learning opportunities across professions. Not only would this *“promote interdisciplinary dialogue and conversation”* around patient care, it would also harness the *“different skills mix of professions”* to build capacity in ante-natal clinics.

Advances in information communication technologies (ICT) were acknowledged as an opportunity to integrate care. The recent pivot to telemedicine and online resources with the COVID-19 pandemic was identified by HCPs as a potential area for growth and expansion to increase the flexibility of ante-natal services and meet patient needs. One midwife suggested: *“Online options, like some videos of gentle exercise sessions the women could do from home. It is a digital world and this may be an opportunity like during COVID to expand our digital or online resources”* (Midwife 12).

## Discussion

The aim of this study was to investigate the perceptions, beliefs and experiences of HCPs who provided routine maternity care for women with PPGP in a metropolitan hospital in Australia. The key message by HCPs interviewed was that the management of PPGP needed to be commenced early during pregnancy and facilitated by an integrated and multidisciplinary approach to ensure comprehensive care.

**Fig. 1:**
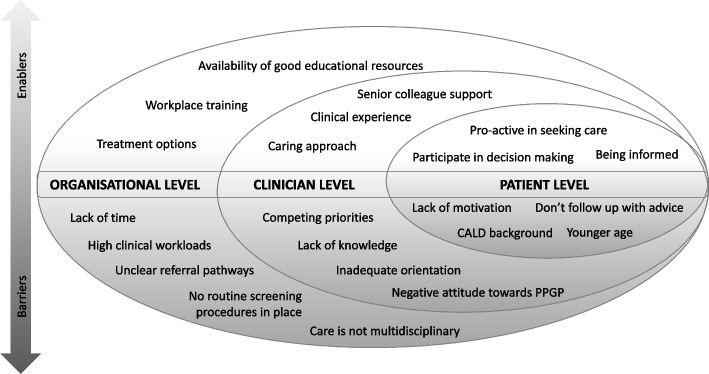
Factors identified as enablers and barriers to care provision for women with PPGP from a patient, clinician and organisational level

Two strong themes that emerged from the interviews were “what works well” and “what gets in the way” regarding current care provision for women with PPGP (Fig. 1). This information depicts perceived enablers and barriers to care and may be used by HCPs and health organisations as a road map to inform improvements in ante-natal care services. Presenting the information from an inner individual patient level, a middle clinician level and an outer organisation level allows for varying care solutions to be considered. Women with PPGP who were informed and pro-active in seeking care, alongside clinicians with a caring approach who had access to adequate resources and workplace training at an organisation level, were likely to assist with care provision. On the other hand, women with PPGP who missed follow up appointments and clinicians who faced competing priorities in an organisational setting which lacked screening procedures and a clear referral pathway, may impede care delivery. This information represented the views of HCPs and provides a novel perspective of health care services towards PPGP.

Situated within the CSM theory, the current study revealed that PPGP was a common condition causing significant disruption to women during their daily life. Further, HCPs agreed that women who received treatment for PPGP had good outcomes consistent with previous studies [7, 9, 10, 13]. Whilst the presentation of PPGP was acknowledged as prevalent, HCPs did not routinely ask women about this type of pain. Therefore, it is possible that PPGP is under-reported and the care for women in pain is being neglected as has been suggested by other authors [7, 9, 10, 12, 13].

In this study, HCPs reported a lack of screening of women for PPGP in maternity care. Whilst Australian clinical practice guidelines for pregnancy care have highlighted PPGP as a common condition, they do not include screening for PPGP as part of routine practice [30]. There is evidence that early screening and intervention in other conditions during pregnancy, such as gestational diabetes and depression, is effective at improving maternal health outcomes and reducing childbirth complications [31–34]. Consequently, maternal care workers have expressed the need for incorporating routine screening in service pathways for practice change to occur [35]. With the growth in knowledge of risk factors associated with PPGP, the inclusion of early screening in routine care could reduce the burden of disease [1].

The current finding that HCPs may have a lack of knowledge about PPGP is supported by previous studies and presents a barrier to care [14, 15]. Most HCPs in this study reported their knowledge about PPGP had developed from on-the-job training and resources, with very few having attended external continuing education courses. Whilst there was mention of the need for training and education to assist new staff, HCPs reported that they felt more confident caring for women with PPGP with experience. Indeed, experience was perceived by all professionals in this study as an important factor in delivering comprehensive care. Nevertheless, the findings support the need to ensure on-boarding procedures include information about PPGP to prepare HCPs who care for pregnant women.

When viewed through the CSM lens, the current study adds to the body of evidence that perceptions of HCPs towards PPGP affect care provision in this population [9, 10, 15]. Attitudes of some staff were identified as an obstacle to care, with HCPs conceding PPGP may be disregarded by colleagues who view PPGP as a normal part of pregnancy and do not consider it curable. However, most HCPs acknowledged that PPGP was manageable with appropriate services, highlighting the need for education of maternity staff to promote empathy and support. Such an approach closely aligns with the CCT, which is underpinned by human connections and a culture of caring to improve health outcomes for women with PPGP [21].

High workloads resulting in time constraints were considered a significant barrier to the provision of care in this study. A lack of time for clinical health consultations has previously been identified as a factor in maternity units in Australia and other countries worldwide [36, 37] and may limit the attention provided to women with PPGP [15]. Not only does this impact on the provision of care for patients, but the systems-related problems include a failure to allow clinicians the capability to provide high quality care. Clinicians spoke of having to prioritise the care they provide due to competing demands and not being able to fulfil their roles as HCPs. This may have serious consequences for occupational satisfaction, fatigue, stress, and potentially lead to burnout of HCPs [38].

There was a view amongst the HCPs interviewed that PPGP could be managed more effectively with a stepped approach to care. The first line care would include education and information for all women with PPGP, and whilst this could be delivered by any HCP as part of interdisciplinary care, midwives, often the point of first contact, may be best placed to provide general information. If the pain was severe and disabling, the second line of care would include physiotherapy and/or review by a doctor. Medical review by a doctor was especially important if analgesia was considered as part of the management or there was concern for an underlying medical or obstetric problem. Importantly, a streamlined pathway, including clear referral processes, is required to ensure timely access to the right care for women.

The findings of this study have several implications for health care. As theorised in the CCT, an integrated approach delivering care that is seamless, effective and efficient is vital to meet the whole of a person’s health needs [21]. The current findings support the importance of a multidisciplinary team for “flexibility” and being able to “match options for treatment to care needs” as part of a patient-centred paradigm [39]. A multidisciplinary approach between HCPs has previously been advocated to optimize the management of women with PPGP [10, 15].

It was acknowledged in this study that there are good educational resources available to support women with PPGP, however, greater visibility was required to ensure all HCPs are aware of existing resources. A unified approach to information delivery and standardised education has been previously identified as important needs by pregnant women [10]. From this study, HCPs also considered the availability of culturally sensitive resources in all languages was essential for good healthcare. Targeting vulnerable women, such as those from a CALD background and those of younger age, is vital to maximise opportunities for engagement by these groups, especially if the advice provided is different to socio-cultural norms or expectations.

The findings suggest HCPs may struggle with cultural differences in the care for women with PPGP. The concept of cultural safety obligates HCP to identify and respect individual preferences while providing care during pregnancy [40]. There is some evidence from this study that culturally safe practices may be lacking, impacting the quality of care delivered and reducing the level of satisfaction for both patients and HCPs. A recent scoping review revealed Australian midwives’ understanding of cultural safety differed widely, and training was required to improve how it is translated into midwifery practice [41]. Improving culturally sensitive practices has the potential to promote a more rewarding relationship between women and their HCPs, and may better meet the care needs of women with PPGP.

Harnessing recent developments in information and communication technologies may help meet the need for flexibility and reach more women. Recognized as a potential area for growth in ante-natal care, technological advances may assist with increasing access to education and resources, such as online exercise programs and videos [10]. Evidence from the recent COVID-19 pandemic suggests there is a good uptake of information provided online or via telehealth by pregnant women [42, 43]. Text messaging has also been shown to be feasible and acceptable in electronic screening and information delivery during pregnancy and may present a novel option for implementation of routine screening and education for PPGP [44].

### Strengths and limitations

The methodology for sampling ensured a large cohort of HCPs from various professions and level of experience who were able to provide rich, authentic experiences. However, participants self-selected into the study from a single hospital site and the findings may not generalise to the beliefs and experiences of HCPs in other health services. Moreover, there were few males who volunteered to participate in this study and there may be a gender bias to the themes even though saturation was achieved.

There was strong agreement across the professions for many themes. Although physiotherapists tended to take a more patho-anatomical approach in their description of PPGP, all three professions acknowledged that psychosocial factors influenced PPGP. The use of CSM as a theory worked well for uncovering HCPs beliefs about PPGP and has provided greater understanding about the views across professions which were similar. Opportunities provided by the alliance of HCPs in caring for women with PPGP adds support for the CCT as an approach to improve healthcare delivery for women with PPGP.

This study offered a flexible approach for participation with interviews conducted either in-person or via tele-video. It is not likely the two modes of data collection impacted the interviews as there were no differences evident in the duration of interviews or the resultant codes between in-person and tele-video interviews. Each participant was able to provide information in a private and confidential manner to ensure their responses were a true indication of their beliefs and perceptions. The use of focus groups may have added to the richness of the information, however, were not possible due to social distancing restrictions during the COVID-19 global pandemic. Therefore, it is not known if the information offered in a group setting would have been different to the responses provided in a one-to-one interview, however, may have been useful to triangulate findings.

## Conclusion

The use of the CSM and CCT as lenses to interpret data in this study provides a strong theoretical framework to underpin knowledge about HCP’s beliefs about PPGP and their expectations regarding care provision. The findings have tangible implications for health care services such that a greater recognition is needed about PPGP being a significant health problem. This will in turn afford greater attention by HCPs to manage this disabling condition and highlights the importance of timely access and clear referral pathways as a means to streamline services for women. Although not historically a part of ante-natal services worldwide, a multidisciplinary and integrated model would support a more coordinated approach to better meet the care needs of women with PPGP.

### Supplementary Information

**Additional file 1.** Interview Guide Questions.

## Data Availability

All data generated or analysed during the current study are included in this published article.

## Abbreviations

PPGP: Pregnancy-related pelvic girdle pain
HCP: Healthcare professionals
CSM: Common-Sense Model
CCT: Convergent Care Theory
COREQ: Consolidated criteria for reporting qualitative research
CALD: Culturally and linguistically diverse
COVID-19: Coronavirus disease of 2019
