# Supplementary material for: “We are not there yet”: perceptions, beliefs and experiences of healthcare professionals caring for women with pregnancy-related pelvic girdle pain in Australia
Source: BMC Pregnancy Childbirth. 2023 Sep 21;23:682. doi: 10.1186/s12884-023-06000-x (PMC10512538; doi:10.1186/s12884-023-06000-x)
Supplement: Supplementary file 1 — Additional file 1. Interview Guide Questions. [file 12884_2023_6000_MOESM1_ESM.docx]

Interview Guide Questions

| Questions | Prompts |
| --- | --- |
| How do you see pregnancy-related pelvic girdle pain? | Tell me about your views on pregnancy-related pelvic girdle pain. Can you explain to me what you think about it? Why is this so? What has influenced the way you see this? |
| Is pregnancy-related pelvic girdle pain a common complaint amongst the women you have seen? | From your experiences, what approximate proportion of women would make such a complaint? How do women with this pain commonly present? What symptoms do they report? Can you describe your level of experience in ante-natal care? |
| Can you describe your experiences in dealing with women with pregnancy-related pelvic girdle pain? | What are the common experiences you have had? Have you had any positive experiences? Have you had any negative experiences? |
| Do you feel comfortable providing care to women with pregnancy-related pelvic girdle pain? | Why do you feel this way? Do you feel confident? Tell me about how it makes you feel. Do you seek help from other staff? Who? Do you refer to other staff or services? Who? Where? Can you give examples of what care you provide? |
| In your view, do you feel you have sufficient knowledge about pregnancy-related pelvic girdle pain to be able to provide healthcare for this condition? | Do you feel you are able to deliver good healthcare? Why? How do you see this? What has shaped your knowledge about this condition? |
| Does anything impact upon your ability to provide care to women with pregnancy-related pelvic girdle pain? | Are there barriers to providing care? What gets in the way? Can you describe any system level issues that may impact on care delivery? What about any clinician level issues that may get in the way? Are there any patient level issues? |
| What supports have you received to help you provide care to women with pregnancy-related pelvic girdle pain? | From where? Any supports from midwives, physiotherapists, doctors or other health care professionals? Tell me about the supports. What about any information from courses, professional development opportunities, online resources? Any other sourced of support or information? |
| What other things do you think would help you provide better healthcare to women with pregnancy-related pelvic girdle pain?  What are your expectations regarding healthcare delivery for women with pregnancy-related pelvic girdle pain?  Is there any advice you would give to other health care providers in working with women experiencing pregnancy-related pelvic girdle pain or to the women themselves? | What about more information about the condition? More education? Tell me about what would you like from health care professionals? From the hospital services? From the women themselves?  Who is best placed to provide care to these women? Are existing services adequate? Is anything missing? What does good care look like? Can you describe this to me?  What would you tell them? Do you have any tips you would share? |
